# The possible role of platelet APP processing in the pathophysiology of Alzheimer’s Disease

**DOI:** 10.1192/j.eurpsy.2024.656

**Published:** 2024-08-27

**Authors:** R. Gurrieri, M. G. Carbone, A. Arone, S. Palermo, D. Marazziti, A. Gemignani

**Affiliations:** ^1^Department of Surgical-Medical and Molecular Pathology and Critical Care Medicine, University of Pisa, Pisa; ^2^Division of Psychiatry, Department of Medicine and Surgery, University of Insubria, Varese; ^3^Department of Clinical and Experimental Medicine, University of Pisa, Pisa; ^4^Saint Camillus International University of Health and Medical Sciences, Unicamillus, Roma, Italy

## Abstract

**Introduction:**

Alzheimer’s disease (AD) stands as the most prevalent form of dementia. Alzheimer’s Disease is acknowledged to have a complex origin, a gradual neurodegenerative progression, and a wide-ranging clinical profile marked primarily by progressive memory loss, cognitive decline, and various functional impairments that significantly diminish the quality of life: Key characteristics of AD encompass the presence of amyloid plaques, which are characterized by the pathological accumulation of insoluble β-amyloid (Aβ) aggregates within the brain tissue and blood vessel walls.Several reports have indicated the existence of cerebral abnormalities within platelets in individuals afflicted by AD.

**Objectives:**

The objective of this investigation was to review studies investigating the metabolism of APP in platelets among individuals with AD to identify potential dependable peripheral indicators leading to novel approaches to its management and treatment.

**Methods:**

A systematic review according to the PRISMA guidelines was carried out, by accessing the PubMed database up to June 2023. The authors screened the titles and the abstracts of all the potentially relevant papers on the basis of a strict list of exclusion and inclusion criteria.

**Results:**

A total of thirty-two studies were included. The evidence points towards the observation that AD individuals exhibit various modifications in platelet APP processing when compared to matched healthy controls, that are frequently associated with the severity of cognitive impairment and functional independence. The majority of the evidence supports changes in platelet ADAM-10 activity, β-secretase activity, APP ratio, a state of heightened platelet activation or hyper-responsiveness, and a potential release of platelet APP via vesicular mechanisms, which may ultimately contribute to Aβ production.

**Conclusions:**

Platelets offer a promising peripheral model for detecting and evaluating molecular changes associated with AD, as they hold the potential to provide vital insights into the development of an effective diagnostic tool and open doors to innovative therapeutic approaches.

**Disclosure of Interest:**

None Declared

